# Xanthohumol, a Prenylated Flavonoid from Hops, Induces DNA Damages in Colorectal Cancer Cells and Sensitizes SW480 Cells to the SN38 Chemotherapeutic Agent

**DOI:** 10.3390/cells9040932

**Published:** 2020-04-10

**Authors:** Alessandra Scagliarini, Aline Mathey, Virginie Aires, Dominique Delmas

**Affiliations:** 1 Université de Bourgogne Franche-Comté, F-21000 Dijon, France; alescaglia@gmail.com (A.S.); Aline.Mathey@u-bourgogne.fr (A.M.); virginie.aires02@u-bourgogne.fr (V.A.); 2 INSERM Research Center U1231—Cancer and Adaptive Immune Response Team, Bioactive Molecules and Health Research Group, F-21000 Dijon, France; 3 Centre Anticancéreux Georges François Leclerc, F-21000 Dijon, France

**Keywords:** Xanthohumol, flavonoids, colorectal cancer, chemosensitization, DNA damage

## Abstract

In spite of chemotherapy and systematic screening for people at risk, the mortality rate of colorectal cancer (CRC) remains consistently high, with 600,000 deaths per year. This low success rate in the treatment of CRC results from many failures associated with high resistance and the risk of metastasis. Therefore, in response to these therapeutic failures, new strategies have been under development for several years aimed at increasing the effect of anticancer compounds and/or at reducing their secondary effects on normal cells, thus enabling the host to better withstand chemotherapy. This study highlights that xanthohumol (Xn) concentrations under the IC_50_ values were able to induce apoptosis and to enhance the DNA-damage response (DDR). We demonstrate for the first time that Xn exerts its anticancer activity in models of colon cancer through activation of the ataxia telangiectasia mutated (ATM) pathway. Subsequently, the ability of Xn to restore DNA damage in CRC cells can sensitize them to anticancer agents such as SN38 (7-ethyl-10-hydroxycamptothecin) used in chemotherapy.

## 1. Introduction

In spite of chemotherapy and systematic screening for people at risk, the mortality rate of colorectal cancer (CRC) remains consistently high, with 600,000 deaths per year. The 5-year survival rate ranges from 90% in patients with stage I to 10% in patients with stage IV disease [1]. This low success rate in the treatment of CRC results from many failures associated with high resistance and the risk of metastasis. Indeed, despite the chemotherapeutic treatments used as monotherapy or in combination, as surgery/radiotherapy, and as targeted therapies, the low survival rate is partly explained by the emergence of intrinsic or acquired resistance mechanisms in tumor cells against therapeutic agents. Several major factors are usually described as being involved in the emergence of resistance to chemotherapy that may be linked to the tumor microenvironment, the host, or the cancer cell. Indeed, we have recently been able to show that tumor metabolism could play a preponderant role in the progression of CRC as well as in the emergence of resistance phenomena linked to the accumulation of complex lipids such as phospholipids [2,3]. This particular tumor microenvironment, associated with a dysfunction in the exacerbated tumor lipid metabolism, is then linked to a decrease in immunogenic cell death, resulting in a decrease in survival rates in patients with metastatic CRC [2]. The hosts themselves can play an important role in these therapeutic failures through a dysfunction of metabolic enzymes or overly high toxicity of the anticancer agent, thereby requiring discontinuation of treatment. This is the case with various drugs such as cisplatin (CDDP), which is a highly effective chemotherapeutic agent for a variety of cancers, including CRC, but causes more side effects including genotoxicity, nephrotoxicity, and acute myelotoxicity [4,5], and 5-fluorouracil (5-FU), which can cause undesirable severe toxicities observed in 10–40% of patients (e.g., hematological, digestive, cardiac disorders, and mucositis) [6,7], subsequently limiting their use. The third factor that plays an important role is the cancer cell itself, which can, following chemotherapeutic treatment, initially develop or acquire resistance systems such as the overexpression of efflux transporters, the modulation of phase I and phase II enzymes, alterations in the cell death pathways, or alteration of the DNA-damage pathway, leading to the death of cancer cells [8,9]. This last point is particularly significant in the case of CRC. Indeed, some studies have shown that colon cancer cells resistant to chemotherapeutic drugs cause a huge reduction in DNA double-strand break (DSB) formation compared to sensitive cancer cells [10]. For example, some reports have shown that DNA DSB formation is reduced in colon cancer cell via the use of the anticancer drug SN38, an active metabolite of irinotecan [10,11]. A highly effective strategy to overcome chemotherapy resistance due to a deficiency in the DNA-damage pathway could be the use of molecules capable of restoring this cell death pathway. We and others have previously shown that DNA DSB formation can be restored in CRC by small molecules such as polyphenols [12]. The pretreatment of resistant metastatic colon cancer cells with resveratrol dramatically enhanced SN38-induced growth inhibition through induction of the DNA-damage pathway by resveratrol and its metabolites [12]. This increase in chemosensitivity is important in clinical practice in order to both restore sensitivity to conventional therapies and to decrease drug toxicity, which is often a factor limiting the use of these anticancer agents.

Therefore, numerous new anticancer drugs have been developed over the last couple of decades, and the demand for a new class of anticancer agents is still high due to the increasing number of drug-resistance cases and serious side effects. One of the most promising sources of new antiproliferative drugs is the plant kingdom, given that data from the Food and Drug Administration (FDA) showed that 40% of the approved molecules are natural compounds or inspired by them, of which 74% are used in anticancer therapy. Therefore, we and others have shown that the use of natural molecules such as polyphenols can modulate xenobiotic-metabolizing enzymes as well as the pathways leading to cancer cell death, making it possible to restore sensitization to anticancer agents. For example, resveratrol, curcumin, vitamin E, and gallic acid are able to protect cells from cisplatin-induced genotoxicity [13,14,15]. Very interestingly, some of these natural compounds are able to synergize at the same time with various anticancer drugs. For example, we have shown that resveratrol, a polyphenol of grapevine and its metabolite, were able to sensitize various colon cancer cells to anticancer drugs, including 5-FU, oxaliplatin, and SN38, via cell cycle disruption, induction of DNA damage, and/or the apoptotic process [12,16].

**Figure 1:**
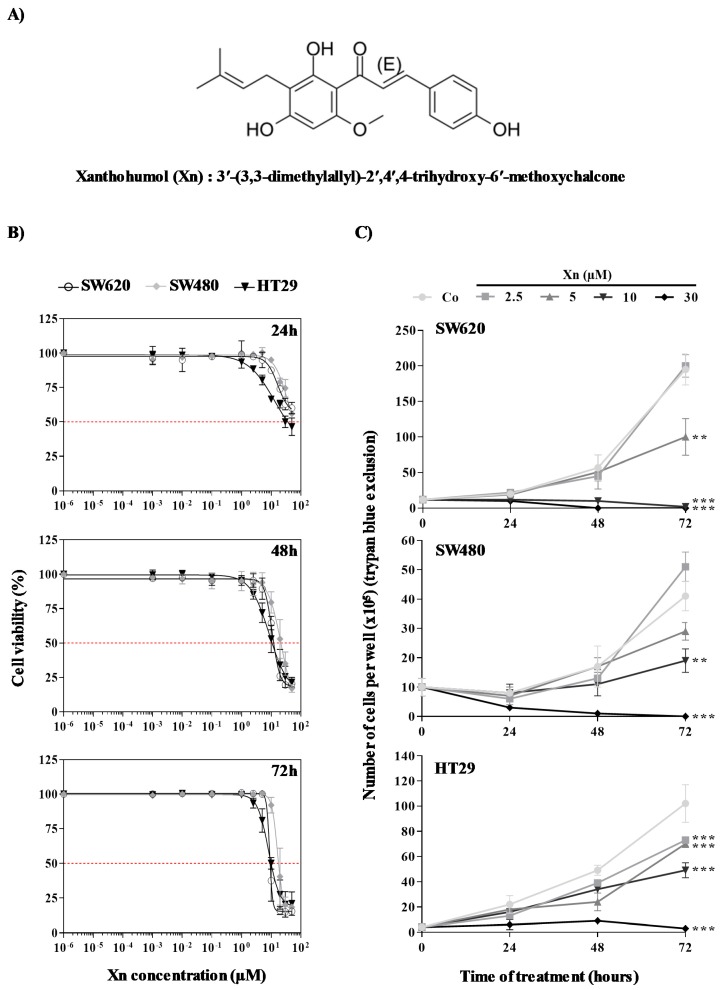
Xanthohumol (Xn) action on colon cancer proliferation and viability. (**A**) Chemical structure of Xn: 3′-(3,3-dimethylallyl)-2′,4′,4-trihydroxy-6′-methoxychalcone. (**B**) After treatment of SW620, SW480, and HT29 cells with increasing Xn concentrations (0–50 μM) at 37 °C for 24, 48, and 72 h, the percentage of cell viability was determined by crystal violet assay. Results are expressed as mean percentage of control growth ± SD of three independent experiments with *n = 6*. (**C**) After 24 h of culture, colon cancer cells SW620, SW480, and HT29 were treated with medium containing vehicle control (0.1% dimethyl sulfoxide (DMSO); Co) or increasing concentrations of Xn. Treated and control cells were harvested at 0, 24, 48, and 72 h. Cell proliferation was quantified with a hemocytometer. Viable cells were distinguished by trypan blue exclusion. The data are mean ± SD of three independent experiments with *n* = 6. *p* values were determined by one-way ANOVA followed by Tukey’s multiple comparison test. * *p* < 0.05, ** *p* < 0.01, and *** *p* < 0.001.

Among the potential compounds that could participate in this promising approach to counteract chemoresistance, a prenylated flavonoid isolated from hops (*Humulus lupulus* L.), xanthohumol (Xn), could be a good candidate (Figure 1A). This prenylated chalcone, 3′-(3,3-dimethylallyl)-2′,4′,4-trihydroxy-6′-methoxychalcone, is the most abundant polyphenol with 0.1–1% of dry weight in hops and can be isolated from the female inflorescence [17]. A number of recent reports have shown that Xn could exert anticancer activities against various cancers such as leukemia [18], hepatocellular carcinoma [19], breast cancer [20,21], prostate cancer [22], colon cancer [23], and ovarian cancer [24]. This anticancer activity involves pleiotropic action on various signaling pathways, such as mitogen-activated protein kinase (MAPK) [25], mitochondria- and Bcl-2-related proteins (intrinsic apoptosis pathway) and the ligation of death receptors belonging to the tumor necrosis factor (TNF)-receptor superfamily (extrinsic apoptosis pathways) [26], and angiogenesis inhibition via the nuclear factor kappa B (NF-κB) pathway [27]. 

Considering the potential of Xn as a chemopreventive agent, we investigated its ability to inhibit the proliferation of three colorectal cell lines and to induce their death by apoptosis. To determine whether Xn exerts a potential adjuvant action with chemotherapeutic drugs, we first analyzed its ability to inhibit the proliferation of three CRC cell lines. Some reports have previously described a potential antiproliferative property for Xn that is highly dependent on cell lines, times of treatment, and concentrations of this prenylated chalcone [19,28,29]. For example, Xn at 10 µM inhibited cell proliferation in the thyroid cancer TPC-1 cell line, supporting a potential action against carcinogenesis, while approximately 100 µM Xn decreased cell viability and the main proapoptotic process [30]. We highlighted that Xn concentrations under the IC_50_ values were able to induce apoptosis and to enhance the DDR. We demonstrated for the first time that Xn exerts its anticancer activity in models of colon cancer by activating the ataxia telangiectasia mutated (ATM) pathway. Subsequently, the ability of Xn to restore DNA damage in CRC cells can sensitize these to anticancer agents such as SN38 (7-ethyl-10-hydroxycamptothecin) used in chemotherapy.

## 2. Materials and Methods

### 2.1. Cell Lines

Human colorectal cancer cell lines SW620, SW480, and HT29 were purchased from the American Type Culture Collection (ATCC, Molsheim, France). SW480 cells are derived from a Duke’s B primary colon adenocarcinoma, and their metastasis-derived counterpart, SW620 cells, are derived from a colorectal adenocarcinoma Duke’s C lymph node metastasis. HT29 cells are derived from a Duke’s C primary colon adenocarcinoma. All cell lines have a microsatellite stable (MSS) phenotype. SW480 and SW620 cells harbor *KRAS*-g12V (substitution-missense) and *TP53* (pR273H; P309S) mutations but are expressing wild-type (wt) *BRAF*, *PIK3CA*, and *PTEN* genes. HT29 cells are wt for *KRAS*, *PI3KCA* and *PTEN* genes but are *BRAF* (V600E) and *TP53* (pR273H) mutated [31]. Cells were maintained in a 5% CO_2_ humidified atmosphere at 37 °C and cultured in Dulbecco’s Modified Eagle’s Medium (DMEM) supplemented with 10% fetal bovine serum (Dutscher, Brumath, France). All cell lines were routinely tested for mycoplasma contamination using the Mycoalert Mycoplasma Detection Kit (Lonza, Levallois-Perret, France).

### 2.2. Reagents and Antibodies

Xanthohumol (Xn) and 7-ethyl-10-hydroxycamptothecin (SN38) were purchased from Sigma-Aldrich (St. Quentin Fallavier, France) and prepared in dimethyl sulfoxide (DMSO). Anti-Cyclin A antibody (sc-751; 1:1000), Cyclin B (sc-752; 1:1000), Cyclin E (sc-481; 1:1000), Cdk1 (sc-54; 1:1000), Cdk2 (sc-6248; 1:500), γH2AX (Ser^139^) (sc-101696; 1:500), and p21 (sc-756; 1:500) were obtained from Santa Cruz (Nanterre, France). Anti-ATM antibody (#2873; 1:1000), p-ATR (Ser^428^) (#2853; 1:1000), and p-p53 (Ser^15^) (#9284; 1:1000) were purchased from Cell Signaling (Ozyme, Saint-Cyr-l’École, France). Anti-p-ATM (Ser^1981^) antibody (ab81292; 1:10000) and p53 (ab131442; 1:500) were obtained from Abcam (Paris, France). Anti-β-actin antibody (#A1978; 1:2000) was obtained from Sigma-Aldrich (St. Quentin Fallavier, France). 

### 2.3. Cell Viability Assays

SW620, SW480, and HT29 cells were seeded into 12-well plates and incubated for 24 h. Cells were then treated with increasing Xn concentrations for 24, 48, and 72 h. Cell viability was first determined using trypan blue staining. The number of viable cells was counted using a KOVA Glasstic Slide (Thermo Fisher Scientific, Illkirch-Graffenstaden, France). Cell viability was also assessed by crystal violet staining (Sigma Aldrich, St. Quentin Fallavier, France). Briefly, cells were seeded into 96-well plates and treated after 24 h of culture. At the end of treatment, cells were washed with phosphate-buffered saline (PBS), fixed with ethanol for 10 min at 4 °C, and then stained with a crystal violet solution (0.5% (*w/v*) crystal violet in 25% (*v/v*) methanol) for 15 min at room temperature. Cells were then gently rinsed with water, and absorbance was measured at 590 nm using a Biochrom Assays UVM 340 microplate reader, following extraction of the dye using an acetic acid 33% solution. The inhibitory concentration 50% (IC_50_), defined as the concentration of Xn that inhibits cell proliferation by 50%, were calculated using a four-parameter nonlinear regression with GraphPad Prism version 6 software (GraphPad Software, La Jolla, San Diego, CA, USA). 

### 2.4. Combination Index Analysis

SW480 cells were seeded into 96-well plates. After overnight incubation, cells were pretreated for 24 h with increasing Xn concentrations (starting concentration, 100 µM; four two-fold serial dilutions). The medium was then removed and replaced with fresh medium containing increasing SN38 concentrations (starting concentration, 25 µM; eight two-fold serial dilutions) for 48 h before crystal violet staining as described above. Drug interactions were quantified using the Chou-Talalay method [32,33]. Thanks to data of drug combination and single-drug inhibition experiments (dose-effect curves), the synergism, additivity, and antagonism of the different combinations of the two drugs were calculated using CompuSyn version 1.0 software (ComboSyn, Inc. Paramus, NJ, USA), on the basis of the multiple drug effect equation and quantification by the combination index (CI). CI was quantified with the following equation: CI = (D)_1_/(D_x_)_1_ + (D)_2_/(D_x_)_2_, where D_1_ and D_2_ represent the doses of drugs 1 and 2 in combination required to achieve the same x% of inhibition as that of drugs 1 (D_x_)_1_ and 2 (D_x_)_2_ when used alone. CI values were generated over a range of Fraction Affected level (FA), indicating x% of inhibited cells, from 0.05 to 0.90. CI > 1, CI = 1, and CI < 1 indicate antagonistic, additive, and synergistic effects, respectively. CI values for each fixed-dose combination of the two drugs were plotted against effect levels (FA). Drug synergy was also assessed by a normalized isobologram, drawn by plotting on both the x- and y-axes the normalization of the dose of each drug (D) in combination (inducing x% inhibition) with the dose at x% inhibition (D_x_) of drugs alone. Combination data points that fell on the line represent an additive interaction, whereas data points below or above the line represent synergism or antagonism, respectively. Besides synergistic combination studies, dose-reduction studies of each drug can be simulated as the inverted terms of the CI equation correspond to the dose-reduction index (DRI), where (DRI)_1_ = (D_x_)_1_/(D)_1_ and (DRI)_2_ = (D_x_)_2_/(D)_2_. (DRI)_1_ (or (DRI)_2_) is defined as the fold of drug 1 (or 2) dose reduction allowed by the synergistic combination while maintaining the same % inhibition as drug 1 (or 2) alone (thereby reducing toxicity). DRI > 1 and DRI < 1 indicate, respectively, favorable and unfavorable dose reduction; DRI = 1 indicates no dose reduction. Similar to the FA-CI plot, the FA-DRI plot is generated using CompuSyn (ComboSyn, Inc., Paramus, NJ 07652, USA). 

### 2.5. Flow Cytometry

All flow cytometry experiments were conducted on a BD FACSCanto^TM^ cytometer equipped with BD FACSDiva software (BD Biosciences, Le Pont de Claix, France), and data were analyzed using FlowJo version 10 software (Tree Star, Ashland, OR, USA).

#### 2.5.1. Cell Cycle Analysis

Cell distribution in cell cycle phases was assessed using the DNA-binding dye propidium iodide (PI). SW620, SW480, and HT29 cells were plated in six-well plates and allowed to recover overnight. Cells were then treated with 2.5, 5, and 10 µM of Xn for 24 and 48 h. Briefly, floating cells and adherent cells were collected, washed with cold PBS, and then fixed/permeabilized with ice-cold 70% ethanol for 24 h at −20 °C. After rehydration in PBS, cells were washed with cold PBS and then incubated with RNase A (200 μg/mL) and PI (50 μg/mL) (Sigma-Aldrich, St. Quentin Fallavier, France) for 1 h at 37 °C in the dark. After staining, cells were washed in cold PBS and the cellular DNA content in the different phases of the cell cycle was then analyzed with the flow cytometer.

#### 2.5.2. Apoptosis Analysis

Cell viability was determined using annexin V-FITC/7-aminoactinomycin D (7AAD) staining from BD Biosciences according to the manufacturer’s instructions. SW620, SW480, and HT29 cells were seeded in 12-well plates and allowed to attach overnight. The following day, cells were treated with 2.5, 5, and 10 µM of Xn for 24 and 48 h. Floating cells and adherent cells were harvested, washed twice with cold PBS, and then incubated with 5 µL of annexin V-FITC and 5 µL of 7AAD in 50 µL of binding buffer for 15 min at room temperature in the dark. At the end of the incubation, 200 μL of binding buffer was added and cells were kept on ice until analysis. Compensation controls included unstained cells and cells stained with either annexin V-FITC or 7AAD alone.

### 2.6. Western Blotting

Cells were seeded into 25-cm^2^ flasks. After 24 h, cells were treated with 2.5, 5, and 10 µM of Xn for 24 and 48 h. Cells were then lysed in radioimmunoprecipitation assay (RIPA) buffer (50 mM Tris-HCl, 150 mM sodium chloride, 0.1% sodium dodecyl sulfate, 0.5% sodium deoxycholate, 1% NP40, and pH8) supplemented with a phosphatase inhibitor, sodium fluoride (50 mM), protease inhibitors such as phenylmethylsulfonyl fluoride (PMSF) (100 μM, Sigma-Aldrich, St. Quentin Fallavier, France), and a protease inhibitor cocktail (Roche, Boulogne-Billancourt, France). Protein quantification was performed using the QuantiPro^TM^ BCA assay kit (Sigma-Aldrich, St. Quentin Fallavier, France). Samples containing 20–60 µg of proteins were prepared in Laemmli gel loading buffer (50 mM Tris-HCl, 10% glycerol, 5% 2-mercaptoethanol, 2% sodium dodecyl sulfate, pH 6.8, and 0.1% bromophenol blue) and then heated for 5 min at 95 °C. Proteins were resolved by sodium dodecyl sulfate–polyacrylamide gel electrophoresis (SDS-PAGE) and transferred to nitrocellulose membranes (Amersham, Les Ulis, France). Blots were then saturated with 5% milk in PBS-Tween 20 0.1% for 1 h at room temperature before overnight incubation at 4 °C with specific primary antibodies. All primary antibodies were diluted in 5% w/v non-fat milk or 5% bovine serum albumin (BSA). Primary antibodies were then detected using appropriate horseradish peroxidase (HRP)-conjugated secondary antibodies (Jackson ImmunoResearch, Interchim, Montlucon, France) for 1 h at room temperature, followed by exposure to enhanced chemiluminescence (ECL) (Bio-Rad, Marnes-la-Coquette, France). A signal was acquired with a ChemiDoc^TM^ XRS + imaging system (Bio-Rad, Marnes-la-Coquette, France), and blots were analyzed with Image Lab^TM^ version 6.0.1 software (Bio-Rad). 

### 2.7. Statistical Analysis

Data are represented as mean ± standard deviation (SD) or standard error of the mean (SEM) of at least three independent experiments. Statistical analysis was conducted using GraphPad Prism version 6 software (GraphPad Software, La Jolla, San Diego, CA, USA). The comparison of continuous data was performed using one-way or two-way ANOVA as appropriate followed by Tukey’s multiple comparison test after having checked data for normal distribution and variance homogeneity. All *p* values are two-tailed; *p* values < 0.05 were considered significant (* *p* < 0.05, ** *p* < 0.01, and *** *p* < 0.001).

## 3. Results 

### 3.1. Antiproliferative Properties of Xanthohumol on Colorectal Cancer Cells

We firstly evaluated the effects of Xn in thee human (SW480, SW620, and HT29) cellular models of CRC with different histological grades. Cells were firstly exposed to increasing concentrations of Xn (0.1–85 µM) for 24, 48, and 72 h prior to cell viability assays (Figure 1B). As shown by crystal violet, which is a nonenzymatic-based assay, since it is only dependent on DNA of live adherent cells, Xn displayed a decrease in cell viability in all cell lines tested in a time- and concentration-dependent manner. It appears that the three CRC cell lines are relatively resistant to Xn after 24 h of treatment up to high concentrations, since we were able to determine the concentration inhibiting 50% of cell viability (IC_50_) for only HT29 cell lines for tested concentrations (Table 1). The percentage of cell viability strongly decreases after 48 h of treatment and, based on IC_50_ determination at 48 and 72 h of treatment (Table 1), SW620 and HT29 cells appeared to be more sensitive to Xn than SW480 cells. Indeed, SW480 cells present an IC_50_ of 10 µM higher than that of the two other cell lines after 48 and 72 h of treatment. Although crystal violet staining remains a reliable, accurate, and rapid method for the indirect estimation of viable cells and the screening of the anticancer activity of compounds, we complemented these experiments by measuring colon cancer cell growth using cell quantification by the trypan blue exclusion test, which is based on the ability of viable cells with an intact membrane to exclude the dye trypan blue by using a hemocytometer in microscopic counting (Figure 1C). For this experiment, we have chosen four concentrations that are below the IC_50_ after 72 h of Xn treatment (2.5, 5, and 10 µM) and one which is above the IC_50_ (30 µM). As previously, 24 h of treatment with Xn are not sufficient to generate significant differences in the number of viable cells; however, we observed that 48 h of Xn treatment induces a significant inhibition of cell proliferation (Figure 1C). Interestingly, Xn induced strong inhibition of cancer cell growth in a time-dependent manner from 5 µM in the three cell lines tested (Figure 1C). However, at a concentration higher than the previously determined IC_50_, such as 30 μM, Xn appeared to be toxic, causing a decrease in the cell number. Therefore, we used nontoxic concentrations for the prenylated chalcone, Xn, for the following experiments, namely 2.5, 5, and 10 µM of Xn.

### 3.2. Xanthohumol Modulates Distribution of Colorectal Cancer Cells in the Cell Cycle

**Figure 2:**
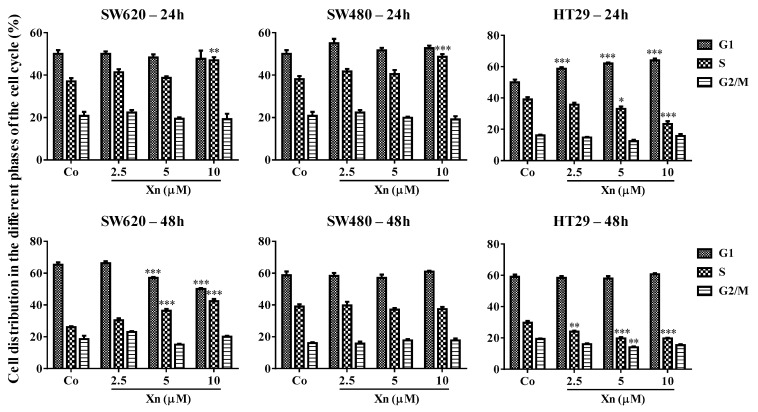
Effects of xanthohumol (Xn) on cell cycle distribution in colon cancer cell lines. After 24 h of culture, colon cancer cells SW620, SW480, and HT29 were treated with medium containing vehicle control (0.1 % DMSO; Co) or increasing concentrations of Xn (2.5, 5, and 10 µM) for 24 and 48 h. Cells were collected and analyzed by flow cytometry after staining with propidium iodide (PI). Histograms represent quantitative analysis of cell distribution in the cell cycle phases (mean ± SD of three independent experiments). *p* values were determined by a two-way ANOVA followed by Tukey’s multiple comparison test. * *p* < 0.05, ** *p* < 0.01, and *** *p* < 0.001.

Usually, tumoral growth cell inhibition is associated with disruption of normal cell cycle progression. Indeed, various studies report that checkpoints at both G1/S and G2/M of the cell cycle are found to be perturbed by phytochemicals [34,35,36] related to their antiproliferative activities. In order to determine whether Xn has the same effect, we analyzed the distribution of CRC cells in the cell cycle by flow cytometry. Colon cancer cells were exposed for 24 and 48 h to three concentrations of Xn (2.5, 5, and 10 µM), for which inhibition of cell proliferation was due to the specific effect of Xn and not due to their toxic effect (Figure 1B,C). As observed previously for cell viability, only SW620 and HT29 present a slight modulation of the distribution of tumor cells within the different phases of the cell cycle after 24 and 48 h of treatment with 5 and 10 μM Xn (Figure 2). Indeed, quantitative analyses of cell distribution in the cell cycle phase show a slight increase in SW620 cells in S phase from 24 h with 5 and 10 µM, which intensifies after 48 h of treatment (Figure 2). This increase in cell number in the DNA replication phase is associated, as investigated, with a decrease in SW620 cells in G1 phase. HT29 cells also show a disturbance in their distribution in the different phases of the cell cycle, but surprisingly, instead of accumulating in the S phase of the cell cycle as for SW620, Xn induces a decrease in HT29 cells in the S phase of the cell cycle after 24 h of treatment, this time being compensated by an increase in cells in phase G1 of the cell cycle (Figure 2). For the cells most resistant to the antiproliferative action of Xn, SW480, it can be seen that there is no change, however slight, in the distribution of cells in the different phases of the cell cycle whatever the treatment time or the concentration used (Figure 2).

### 3.3. Involvement of Cyclins and their Cdks in Xanthohumol-Induced Cell Cycle Disruption

**Figure 3:**
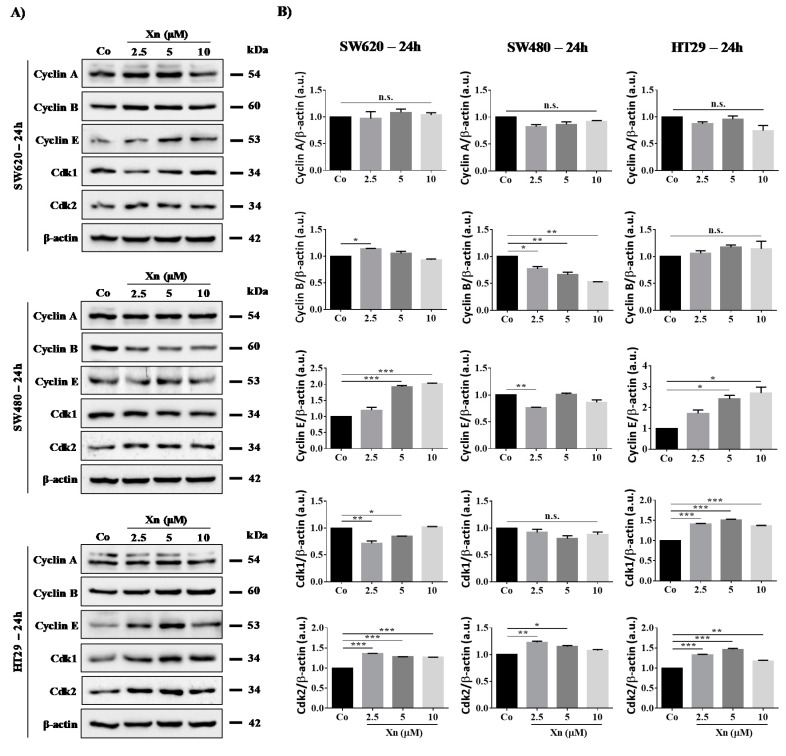
Modulation of key cell cycle regulators after 24 h of treatment with xanthohumol (Xn) in colon cancer cell lines. (**A**) Immunoblot analysis of Cyclin A, B, E, Cdk1, and Cdk2 in Xn (2.5, 5, and 10 µM)-treated SW620, SW480, and HT29 cells for 24 h. β-actin: loading control. (**B**) Densitometry quantification of western blotting obtained in Figure 3A. Data are expressed as mean fold induction ± SEM of three independent experiments. *p* values were determined by a one-way ANOVA followed by Tukey’s multiple comparison test. * *p* < 0.05, ** *p* < 0.01, and *** *p* < 0.001.

**Figure 4:**
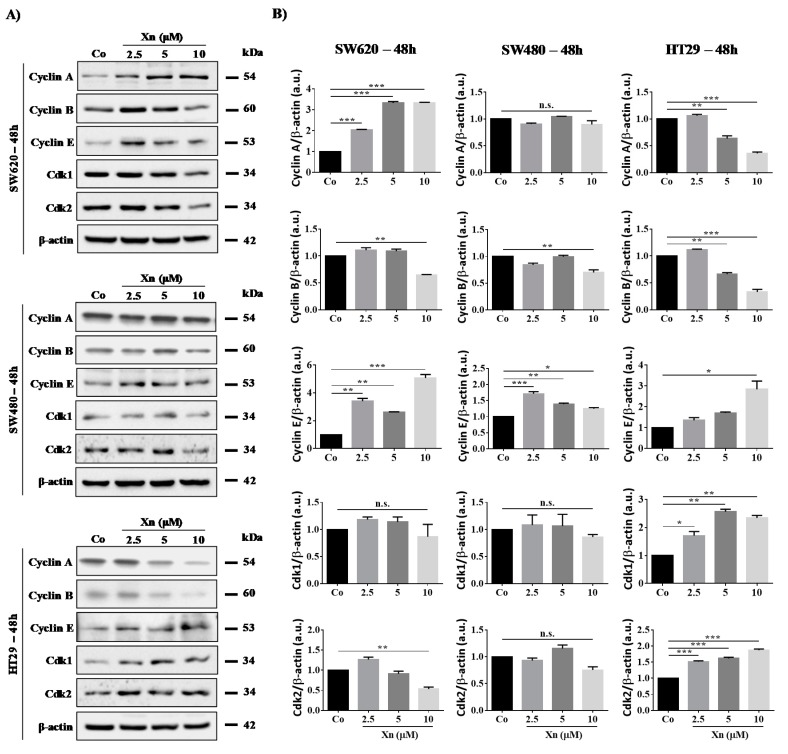
Modulation of key cell cycle regulators after 48 h of treatment with xanthohumol (Xn) in colon cancer cell lines. (**A**) Immunoblot analysis of Cyclin A, B, E, Cdk1, and Cdk2 in Xn (2.5, 5, and 10 µM)-treated SW620, SW480, and HT29 cells for 48 h. β-actin: loading control. (**B**) Densitometry quantification of western blotting obtained in Figure 4A. Data are expressed as mean fold induction ± SEM of three independent experiments. *p* values were determined by a one-way ANOVA followed by Tukey’s multiple comparison test. * *p* < 0.05, ** *p* < 0.01, and *** *p* < 0.001.

Modulation of tumor cell distribution in the different cell cycle phases prompted us to investigate whether the key regulators of cell cycle progression, cyclins and their partners, the cyclin-dependent kinases (Cdks) could be affected upon exposure to Xn. We have previously shown with another natural compound—resveratrol, an emblematic polyphenol—that polyphenol-induced colon cancer cell accumulation in the S phase was associated with an increase in cyclin A and B expression as well as Cdk2 protein levels, which have been shown to be required for DNA synthesis [12,16,37,38]. We observed similar results with Xn, where a slight increase is observed at 24 h of treatment with Xn concerning Cdk2 and cyclin E in SW620 cell line (Figure 3). Then, at 48 h of treatment, Xn increases cyclin A and E expression, whereas cyclin B is slightly decreased at the higher concentration of 10 µM (Figure 4). Consistent with the entry of cells into the S phase, there is usually an increase in nuclear Cdk2 activity associated with both cyclins A and E [16,39]. Conversely, the G0/G1 phase is triggered by the cyclin E that appears at the start of the G1 phase and reaches its maximum level near the G1/S border. Cyclin E associates with Cdk2 or Cdk1 and controls the checkpoint G1/S transition. HT29 cells exposed to increased concentrations of Xn show an increase in cyclin E as well as its associated Cdks, Cdk1 and 2, from 24–48 h of treatment (Figure 3 and Figure 4). In accordance with results of cell viability and cell cycle distribution, the most resistant colon cancer cells, SW480, did not show modulation of the key cell cycle regulators, whether cyclins or Cdks (Figure 3 and Figure 4).

### 3.4. Xanthohumol Induces DNA Damage in Colon Cancer Cells

Various reports have correlated the cell cycle delay and cell growth arrest to the induction of cell death through the induction of DNA damage and an increase in p53 phosphorylation. Both processes are reported to depend on the DNA damage checkpoint kinases ATM/ataxia telangiectasia-Rad3-related (ATR) [40] and checkpoint kinase (Chk) [41]. ATM is a protein that is well known as a central mediator of responses to DNA DSB and subsequent replication stress, whereas the ATR protein is activated in response to stress at the level of replication forks and to DNA single-strand break (SSB) lesions. In all cases, ATM/ATR activation phosphorylates downstream the histone H_2_AX (γ-H_2_AX), which constitutes a recognized marker of DNA damage.

**Figure 5:**
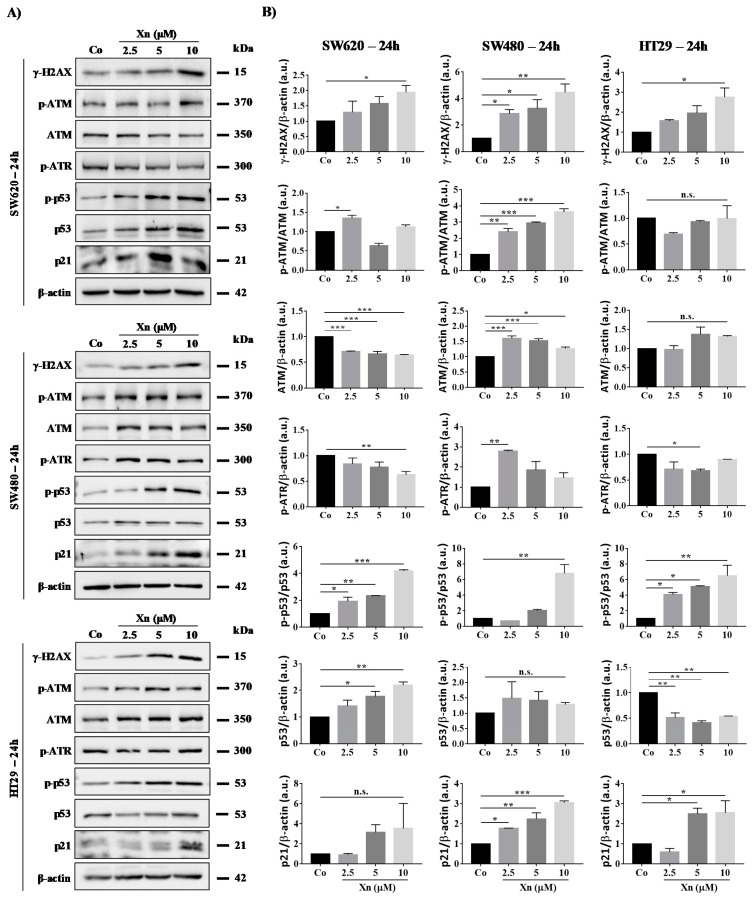
Xanthohumol (Xn) induces a DNA damage pathway in colon cancer cell lines after 24 h of treatment. (**A**) Immunoblotting analysis of phosphorylated H2AX, phospho-ATM, ATM, phospho-ATR, phospho-p53, p53, and p21 in the three colon cancer cell lines SW620, SW480, and HT29. Cells were incubated with increasing concentrations of Xn (0, 2.5, 5, and 10 µM) for 24 h (**A**). β-actin: loading control. One representative example of three independent experiments. (**B**) Densitometry quantification of western blotting obtained in Figure 5A. Data are expressed as mean fold induction ± SEM of three independent experiments. *p* values were determined by a one-way ANOVA followed by Tukey’s multiple comparison test. * *p* < 0.05, ** *p* < 0.01, and *** *p* < 0.001.

**Figure 6:**
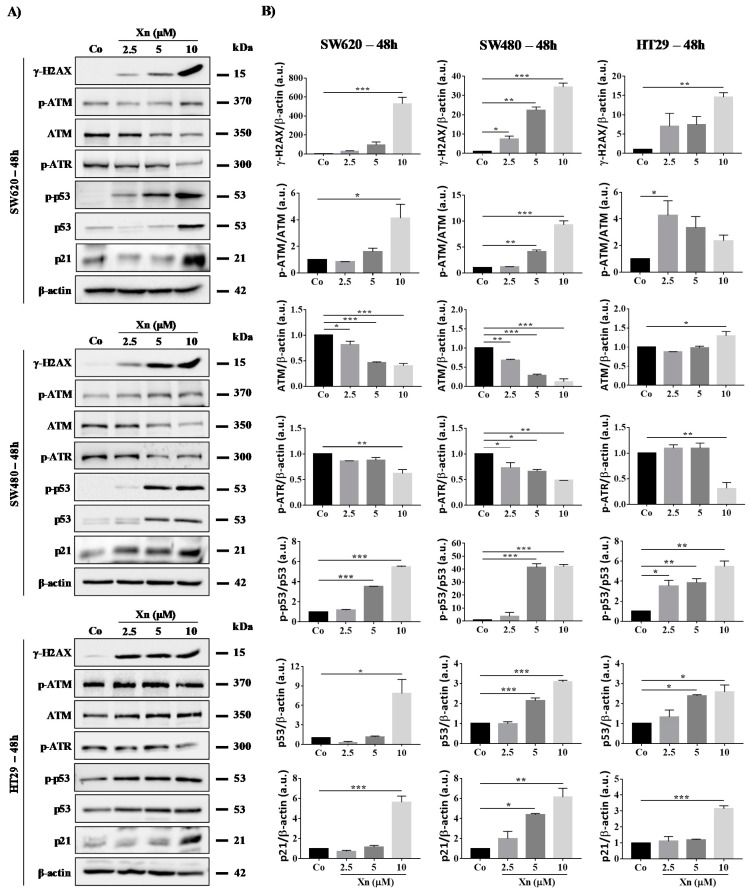
Xanthohumol (Xn) induces a DNA damage pathway in colon cancer cell lines after 48 h of treatment. (**A**) Immunoblotting analysis of phosphorylated H2AX, phospho-ATM, ATM, phospho-ATR, phospho-p53, p53, and p21 in the three colon cancer cell lines SW620, SW480, and HT29. Cells were incubated with increasing concentrations of Xn (0, 2.5, 5, and 10 µM) for 48 h. β-actin: loading control. One representative example of three independent experiments. (**B**) Densitometry quantification of western blotting obtained in Figure 6A. Data are expressed as mean fold induction ± SEM of three independent experiments. *p* values were determined by a one-way ANOVA followed by Tukey’s multiple comparison test. * *p* < 0.05, ** *p* < 0.01, and *** *p* < 0.001.

In all colon cancer cell lines tested, Xn at very low concentrations of 2.5–10 µM induces a strong increase in phosphorylated-histone H_2_AX (γ-H_2_AX), which reflects DNA damage levels at 24 h (Figure 5) and 48 h (Figure 6). Very interestingly, SW480, which is a more resistant cell line, exhibits strong induction of H_2_AX serine-19 phosphorylation from 2.5 to 10 µM Xn, which could be associated with apoptosis of the lethally damaged cells. Subsequently, the DDR pathway induced by the activation of ATM/ATR proteins is modulated in colon cancer cells. Indeed, Xn induces ATM activation through its phosphorylation, which is surprisingly more important in SW480 cells compared to SW620 and HT29 cells (Figure 5 and Figure 6). This enhancement of the phosphorylated form of ATM is associated with a decrease in ATM protein at 48 h (Figure 6). Compared to SW620 and HT29 cells, only in SW480 cells was Xn able to induce phosphorylation of both ATM and ATR at 24 h of treatment (Figure 5 and Figure 6), suggesting that this prenylated chalcone could lead to DSB as well as SSB in this tumoral cell line. In all cases, whatever the form initially activated, the consequence of these activations is p53 activation, which has been shown to respond to DNA damage by an increase in its phosphorylation at serine-20 (Figure 5 and Figure 6). In agreement with the activation of p53, we observed that Xn induced p21 expression, which has been described as one of the transcriptional targets of the tumor suppressor protein p53 (Figure 5 and Figure 6).

### 3.5. Xanthohumol Induces Apoptosis in Colon Cancer Cells

**Figure 7:**
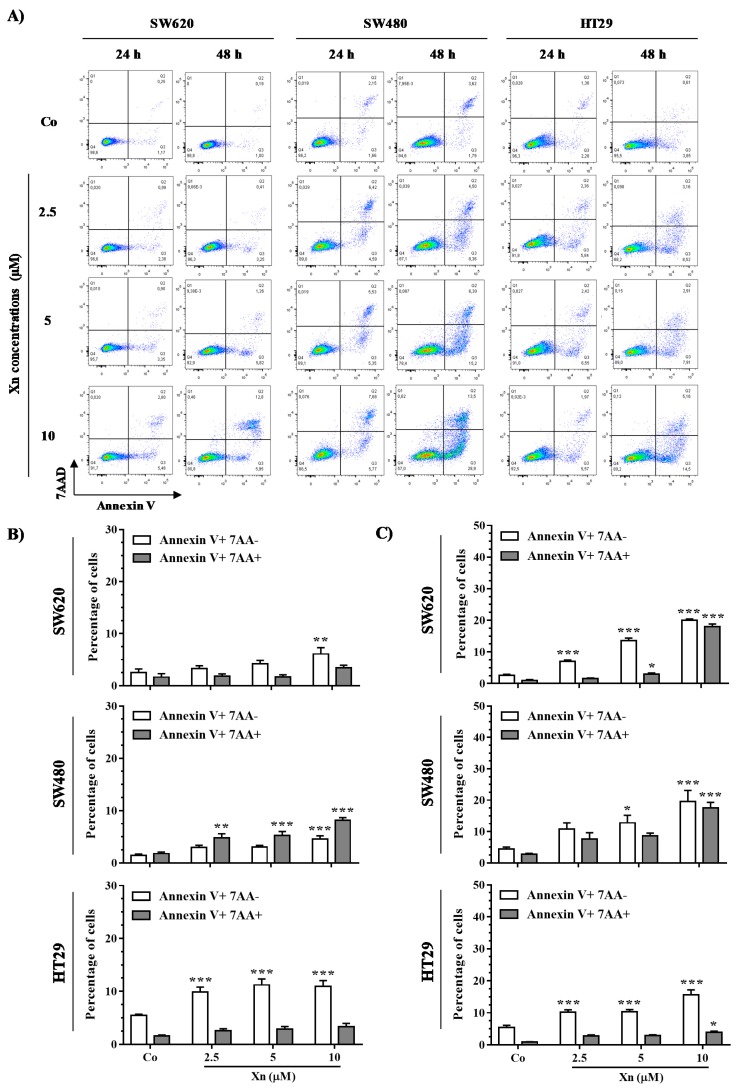
Xanthohumol (Xn) induces apoptosis of colon cancer cells. After 24 h of culture, colon cancer cells SW620, SW480, and HT29 were treated with medium containing vehicle control (0.1% DMSO; Co) or increasing concentrations of Xn (2.5, 5, and 10 µM) for 24 and 48 h and then stained with Annexin V/7-aminoactinomycin D (7AAD) at the end of treatment. (**A**) Representative Annexin V/7AAD flow cytometry dot plots, obtained from colorectal cancer cells treated with the indicated Xn concentrations for 24 and 48 h. The percentages of early apoptotic cells (identified as the Annexin V-positive/7AAD-negative population, in white bar) and late apoptotic cells (identified as the Annexin V-positive/7AAD-positive population, grey bar) were calculated, after 24 h (**B**) and 48 h (**C**) of treatment with the indicated Xn concentrations. The data are means ± SEM of three independent experiments. *p* values were determined by a two-way ANOVA followed by Tukey’s multiple comparison test. * *p* < 0.05, ** *p* < 0.01, and *** *p* < 0.001.

Following DNA damage, cells either repair the damage or undergo apoptotic death, mostly in a p53-dependent manner. To clarify how colon cancer cells respond to DNA damage, we investigated Xn-induced cell death. Colon cancer cells were double stained with annexin V/7AAD after treatment with Xn at 2.5, 5, and 10 µM for 24 h and 48 h. It appears that Xn induces an increase of global level of apoptosis in the three tested colorectal cancer cell lines as compared to the control (Figure 7). More particularly, the level of early apoptosis is more important than late apoptosis in HT29 cells, which starts from 24 h of treatment in a concentration-dependent manner compared to the control (Figure 7A,B). 

### 3.6. Xanthohumol Synergized with SN38 to Inhibit SW480 Colorectal Cancer Cell Proliferation

**Figure 8:**
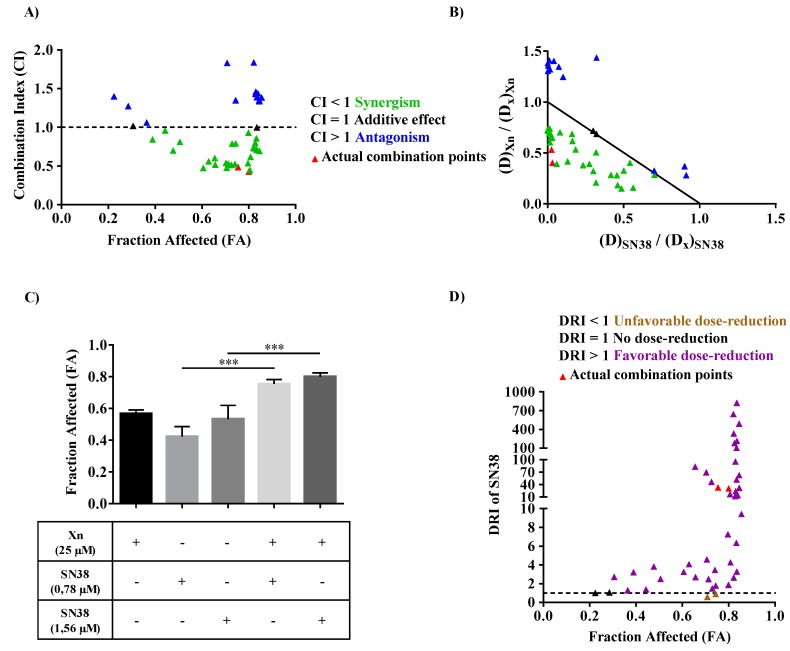
Determination of the combinatory effect of xanthohumol (Xn) with SN38 on SW480 colon cancer cell viability. SW480 cells were pretreated with increasing Xn concentrations for 24 h. The medium was then removed and replaced with fresh medium containing increasing SN38 concentrations for the last 48 h. The amounts of viable cells were normalized with vehicle control after crystal violet staining. Dose-effect curves of drugs alone and in combination were then simulated using CompuSyn software. (**A**) Fraction Affected (FA)-Combination Index (CI) plot. The data presented are the combined results of four independent experiments. CI < 1, CI = 1, and CI > 1 indicate synergistic, additive, and antagonistic effects, respectively. The horizontal dash line corresponds to CI = 1. (**B**) Normalized isobologram with normalization of the dose of each drug (D) in combination (inducing x% inhibition) with the dose at x% inhibition (D_x_) of drugs alone on both the x- and y-axes. Combination data points on the diagonal line indicates additive effects, on the lower left indicates synergism, and on the upper right indicates antagonism. (**C**) Growth inhibition efficacy (or FA) for the actual synergistic combinations described as red triangles in Figure 8A,B,D. Data are represented as means ± SD of four independent experiments. *p* values were determined by a one-way ANOVA followed by Tukey’s multiple comparison test. *** *p* < 0.001. (**D**) FA-dose reduction index (DRI) plot of SN38. DRI > 1 and DRI < 1 indicate favorable and not favorable dose reduction; DRI = 1 indicates no dose reduction. The horizontal dash line at DRI = 1 was drawn. The data are representative of four independent experiments.

In order to assess the potential use of Xn as a chemosensitizing agent able to reduce drug resistance, we studied the potential synergistic effect of Xn with SN38, a widely used chemotherapeutic drug for colorectal cancer. SW480 cells were thus pretreated for 24 h with increasing Xn concentrations. The medium was then removed and replaced with fresh medium containing increasing SN38 concentrations for 48 h before cell viability assay using crystal violet staining. Interactions between these two drugs were then analyzed by determining the combination index (CI) thanks to the Chou–Talalay equation, where CI < 1 indicates synergism, CI = 1 shows additivity, and CI > 1 antagonism. Afterwards, CI for all combination data points were calculated and plotted against Fraction Affected (FA), defined as growth inhibition potency. The normalized isobologram, characterized by different dose combinations of Xn and SN38 normalized with the dose of each drug alone, at a given x % growth inhibition, also allow to distinguish between synergism, additivity and antagonism since each dot is defined as a CI value. Most of the drug combinations show synergistic effect (green dots; CI < 1; Figure 8A), especially those reducing cell viability from 60% to 80% (0.6 < FA < 0.8) (Figure 8A), as well as the synergistic combinations plotted in normalized isobologram (Figure 8B). Among these synergistic combinations, the one corresponding to 25 μM Xn with either 0.78 or 1.56 μM SN38 (red triangles, defined as “actual combination points”, Figure 8A,B) was associated with a strong reduction of cancer cell viability (corresponding to high FA values) as compared to conventional agents alone (Figure 8C). Thus, Xn could be used as a potential chemosensitizer to enhance anticancer properties of SN38. Since reducing the dose of synergistic combinations at high effect levels is more relevant to anticancer therapies, the dose-reduction index (DRI) of SN38 was then calculated by inverting the terms of the Chou-Talalay equation which defines the combination index. Under exposure of Xn, the dose of SN38 can be reduced (almost all DRI > 1; Figure 8D), including those of synergistic combinations that induce high effect levels (red triangles, Figure 8D). Therefore, this would allow to reduce toxicity and side effects in anticancer therapy while maintaining drug efficacy.

## 4. Discussion

In spite of chemotherapy and systematic screening for people at risk, the mortality rate of CRC has remained consistently high over the last 10 years. This low success rate in the treatment of CRC results from many failures associated with high resistance and the risk of metastasis. Therefore, in response to these therapeutic failures, new strategies have been under development for several years, aimed at increasing the effect of anticancer compounds and/or reducing their secondary effects on normal cells, thus enabling the host to better withstand chemotherapy. The present study highlights the ability of a prenylated flavonoid, Xn, to induce activation of DDR and its cellular consequences, in particular apoptosis. This induction of DNA damage by Xn constitutes a very interesting property to sensitize resistant colon cancer cells such as SW480 to classical anticancer drugs. Indeed, we found for the first time that Xn was able to cooperate positively with a metabolite of irinotecan, SN38, that could potentially enhance DNA damage in SW480 that usually exhibits a huge reduction in DNA DSB formation [10]. Synergistic combinations of Xn with SN38 leads to an increase in SW480 cell death and has the benefit of potentially decreasing the concentration of SN38, as revealed by the DRI values of SN38 (Figure 8). 

The human genome is subject to permanent stress of exogenous (e.g., ionizing radiation and ultraviolet) or endogenous origin (e.g., reactive oxygen species and replication errors), which can cause lesions of varying nature, such as DNA DSB or DNA SSB. In normal cells, these lesions are detected at cell cycle checkpoints and supported by a complex network of signaling pathways commonly known as DDR. Depending on the severity of the lesions induced, DDR activates either DNA repair systems, thus allowing the resumption of cell proliferation and survival, or activates apoptosis or senescence pathways in order to maintain global genome integrity and prevent oncogenesis [42]. Genomic instability and defects in DDR (due to mutations of key molecular players) are considered as one of the characteristics of tumor cells [43]. These are not only associated with the accumulation of mutations induced in particular by the accumulation of reactive oxygen species but are also responsible for tumor cell resistance to radiotherapy and conventional anticancer drugs targeting DDR [44]. This resistance is partly linked to prolonged activation of DDR, which can reactivate DNA repair systems and therefore promote the survival, proliferation, and dissemination of malignant cells that were not initially targeted by anticancer agents [45]. The major pathways involved in DDR are the ATM and ATR pathways. ATM is specifically activated in response to DSB-type lesions, which are the most dangerous for the cell, while the ATR protein is activated in response to stress at the level of the replication forks and SSB-type lesions. Both ATM and ATR proteins phosphorylate histone H2AX (γ-H2AX) downstream, which subsequently recruits other substrates of ATM and ATR. The latter modulates the activity of key regulators of the cell cycle through activation of the transcription factor p53 and its target genes such as the p21 gene encoding the p21 protein. The latter makes it possible to trigger the G1/S, S, and G2/M checkpoints and thus to block progression of the cell cycle in order to allow DNA repair by specific enzymes, such as poly (ADP-ribose) polymerase (PARP) protein, or to induce cell death [42,46]. Very interestingly, we found that Xn was able to induce the activation of DNA damage pathways in all colon cancer cell lines but to modulate differentially the distribution of these tumor cells in the different phases of the cell cycle. The two most Xn-sensitive colon cancer cells, SW620 and HT29, both show disruption of the cell cycle in the G0/G1 and S phase, as revealed by analyzing cell distribution in phases and the key regulators of cell cycle, which are cyclins and their kinases, Cdks. We confirmed the results previously obtained by Liu et al., showing a depletion of cyclin B1 in HT29 [47], and by Logan et al., showing an arrest in the G0/G1 phase [48]. The differing results from these two authors probably result from the different concentrations used on this same HT29 line [47,48]. Here, we show that nontoxic concentrations of Xn (2.5–10 µM) were able to decrease key cyclins, such as cyclins A and B, and conversely to increase cyclin E, which controls checkpoint G1/S transition when associated with its cyclin-dependent kinases Cdk1 and 2, both of which are greatly increased depending on treatment time and concentrations (Figure 3 and Figure 4). Very interestingly, Xn-induced cell cycle disruption is not comparable in metastatic colorectal SW620 cells, where Xn preferentially increases the SW620 cell DNA replication phase, which is again confirmed by analysis of the proteins controlling the checkpoint. Indeed, cyclin A and E protein expression is increased, whereas cyclin B is slightly decreased at the higher concentration of 10 µM (Figure 4B). Conversely, the most resistant colon cancer cells, SW480, did no show disruption in cell cycle progression but significant apoptosis after 48 h of treatment with Xn. Besides, Xn can strongly sensitize SW480 cells to the action of a classical drug such as SN38. We demonstrated that Xn was able to activate p53, which is one of the transcriptional targets, and p21, which plays a key role in DNA damage. This is a crucial point, since the use of Xn pretreatment may not only improve the efficacy of classical chemotherapeutic drugs but also help in reducing their dose and thus in reducing their toxicity, as shown by the DRI determination of SN38 (Figure 8D). This point is relevant, since the use of SN38 is often associated with severe gastrointestinal toxicities and neutropenia, thereby limiting its widespread use [49]. Although our combination experiments between Xn and SN38, performed in a sequential scheme (cells were pretreated for 24h Xn before the addition of SN38), displayed synergism interactions (Figure 8), the study of Ambroz et al. nicely showed in colorectal cancer cells lines (i.e., SW480, SW620, and Caco-2) that cotreatment of cells with hop-derived prenylflavonoids including Xn with chemotherapies such as 5-Fluorouracil, oxaliplatin, and irinotecan (SN38 being an irinotecan-derived potent metabolite), mostly resulted in antagonism interactions [50]. Hence, combined observations from the present study and that from Ambroz et al. suggest that synergy between Xn and chemotherapy drugs can only fully take place when drugs are sequentially administered and that simultaneous administration should be avoided because it tremendously reduces anticancer drug efficacy.

## 5. Conclusions

To date, Xn, a natural prenylated flavonoid from hops, has been described as having antioxidant [51], antiviral and antibacterial [52], antidiabetic [53], anti-inflammatory [54], and anticancer activities [55]. These properties have made it possible to classify this chalcone in the category of chemopreventive molecules. Moreover, a number of studies have been able to show the ability of Xn to prevent DNA damage induced by numerous cytotoxic agents [56,57,58]. This study highlights for the first time the ability of Xn to sensitize colon cancer cells to an anticancer agent such as SN38. This ability is associated in this case with the ability of Xn to induce lethal DNA damage and its signaling pathways. Therefore, additional studies must now be conducted with a wider panel of different anticancer drugs and cancer cell lines in order to better understand the chemosensitizing action of Xn and to investigate whether this prenylated chalcone can be classed as a potential adjuvant to increase sensitivity to anticancer agents.

## Figures and Tables

**Table 1:** Inhibitory concentration 50% (IC_50_) values determined from cell viability assays for xanthohumol on the colon cancer cell lines at the different times of treatment.

	IC_50_	24 h	48 h	72 h
Cell lines	SW620	-	12 ± 3.57 µM	7 ± 1.38 µM
	SW480	-	22 ± 6.49 µM ^$^	20 ± 3.30 µM ^££^
	HT29	39 ± 6.48 µM ***	12 ± 2.80 µM	10 ± 1.75 µM

*p* values were determined by one-way ANOVA followed by Tukey’s multiple comparison test. *** *p* < 0.001, HT29 vs SW620 and SW480; $ *p* < 0.05, SW480 vs HT29; and ££ *p* < 0.01, SW480 vs HT29 and SW620.

## References

[1] Brenner H., Kloor M., Pox C.P. (2013). Colorectal cancer. Lancet.

[2] Cotte A.K., Aires V., Fredon M., Limagne E., Derangere V., Thibaudin M., Humblin E., Scagliarini A., De Barros J.P., Hillon P. (2018). Lysophosphatidylcholine acyltransferase 2-mediated lipid droplet production supports colorectal cancer chemoresistance. Nat. Commun..

[3] Cotte A.K., Cottet V., Aires V., Mouillot T., Rizk M., Vinault S., Binquet C., De Barros J.P., Hillon P., Delmas D. (2019). Phospholipid profiles and hepatocellular carcinoma risk and prognosis in cirrhotic patients. Oncotarget.

[4] Makovec T. (2019). Cisplatin and beyond: Molecular mechanisms of action and drug resistance development in cancer chemotherapy. Radiol. Oncol..

[5] Ozkok A., Edelstein C.L. (2014). Pathophysiology of cisplatin-induced acute kidney injury. BioMed Res. Int..

[6] Allison J.D., Tanavin T., Yang Y., Birnbaum G., Khalid U. (2020). Various Manifestations of 5-Fluorouracil Cardiotoxicity: A Multicenter Case Series and Review of Literature. Cardiovasc. Toxicol..

[7] Tsalic M., Bar-Sela G., Beny A., Visel B., Haim N. (2003). Severe toxicity related to the 5-fluorouracil/leucovorin combination (the Mayo Clinic regimen): A prospective study in colorectal cancer patients. Am. J. Clin. Oncol..

[8] Aires V., Colin D.J., Doreau A., Di Pietro A., Heydel J.M., Artur Y., Latruffe N., Delmas D. (2019). P-Glycoprotein 1 Affects Chemoactivities of Resveratrol against Human Colorectal Cancer Cells. Nutrients.

[9] Delmas D., Solary E., Latruffe N. (2011). Resveratrol, a phytochemical inducer of multiple cell death pathways: Apoptosis, autophagy and mitotic catastrophe. Curr. Med. Chem..

[10] Gongora C., Vezzio-Vie N., Tuduri S., Denis V., Causse A., Auzanneau C., Collod-Beroud G., Coquelle A., Pasero P., Pourquier P. (2011). New Topoisomerase I mutations are associated with resistance to camptothecin. Mol. Cancer.

[11] Petitprez A., Poindessous V., Ouaret D., Regairaz M., Bastian G., Guerin E., Escargueil A.E., Larsen A.K. (2013). Acquired irinotecan resistance is accompanied by stable modifications of cell cycle dynamics independent of MSI status. Int. J. Oncol..

[12] Aires V., Limagne E., Cotte A.K., Latruffe N., Ghiringhelli F., Delmas D. (2013). Resveratrol metabolites inhibit human metastatic colon cancer cells progression and synergize with chemotherapeutic drugs to induce cell death. Mol. Nutr. Food Res..

[13] Al-Eitan L.N., Alzoubi K.H., Al-Smadi L.I., Khabour O.F. (2020). Vitamin E protects against cisplatin-induced genotoxicity in human lymphocytes. Toxicol. In Vitro.

[14] Shruthi S., Bhasker Shenoy K. (2018). Genoprotective effects of gallic acid against cisplatin induced genotoxicity in bone marrow cells of mice. Toxicol. Res..

[15] Wang Y., Hu P.C., Gao F.F., Lv J.W., Xu S., Kuang C.C., Wei L., Zhang J.W. (2014). The protective effect of curcumin on hepatotoxicity and ultrastructural damage induced by cisplatin. Ultrastruct. Pathol..

[16] Colin D., Gimazane A., Lizard G., Izard J.C., Solary E., Latruffe N., Delmas D. (2009). Effects of resveratrol analogs on cell cycle progression, cell cycle associated proteins and 5fluoro-uracil sensitivity in human derived colon cancer cells. Int. J. Cancer.

[17] Nikolic D., van Breemen R.B. (2013). Analytical methods for quantitation of prenylated flavonoids from hops. Curr. Anal. Chem..

[18] Benelli R., Vene R., Ciarlo M., Carlone S., Barbieri O., Ferrari N. (2012). The AKT/NF-kappaB inhibitor xanthohumol is a potent anti-lymphocytic leukemia drug overcoming chemoresistance and cell infiltration. Biochem. Pharmacol..

[19] Ho Y.C., Liu C.H., Chen C.N., Duan K.J., Lin M.T. (2008). Inhibitory effects of xanthohumol from hops (Humulus lupulus L.) on human hepatocellular carcinoma cell lines. Phytother. Res..

[20] Yoo Y.B., Park K.S., Kim J.B., Kang H.J., Yang J.H., Lee E.K., Kim H.Y. (2014). Xanthohumol inhibits cellular proliferation in a breast cancer cell line (MDA-MB231) through an intrinsic mitochondrial-dependent pathway. Indian J. Cancer.

[21] Monteiro R., Calhau C., Silva A.O., Pinheiro-Silva S., Guerreiro S., Gartner F., Azevedo I., Soares R. (2008). Xanthohumol inhibits inflammatory factor production and angiogenesis in breast cancer xenografts. J. Cell. Biochem..

[22] Deeb D., Gao X., Jiang H., Arbab A.S., Dulchavsky S.A., Gautam S.C. (2010). Growth inhibitory and apoptosis-inducing effects of xanthohumol, a prenylated chalone present in hops, in human prostate cancer cells. Anticancer Res..

[23] Miranda C.L., Stevens J.F., Helmrich A., Henderson M.C., Rodriguez R.J., Yang Y.H., Deinzer M.L., Barnes D.W., Buhler D.R. (1999). Antiproliferative and cytotoxic effects of prenylated flavonoids from hops (Humulus lupulus) in human cancer cell lines. Food Chem. Toxicol..

[24] Drenzek J.G., Seiler N.L., Jaskula-Sztul R., Rausch M.M., Rose S.L. (2011). Xanthohumol decreases Notch1 expression and cell growth by cell cycle arrest and induction of apoptosis in epithelial ovarian cancer cell lines. Gynecol. Oncol..

[25] Festa M., Capasso A., D’Acunto C.W., Masullo M., Rossi A.G., Pizza C., Piacente S. (2011). Xanthohumol induces apoptosis in human malignant glioblastoma cells by increasing reactive oxygen species and activating MAPK pathways. J. Nat. Prod..

[26] Pan L., Becker H., Gerhauser C. (2005). Xanthohumol induces apoptosis in cultured 40-16 human colon cancer cells by activation of the death receptor- and mitochondrial pathway. Mol. Nutr. Food Res..

[27] Saito K., Matsuo Y., Imafuji H., Okubo T., Maeda Y., Sato T., Shamoto T., Tsuboi K., Morimoto M., Takahashi H. (2018). Xanthohumol inhibits angiogenesis by suppressing nuclear factor-kappaB activation in pancreatic cancer. Cancer Sci..

[28] Liu W., Li W., Liu H., Yu X. (2019). Xanthohumol inhibits colorectal cancer cells via downregulation of Hexokinases II-mediated glycolysis. Int. J. Biol. Sci..

[29] Liu X., Song M., Wang P., Zhao R., Chen H., Zhang M., Shi Y., Liu K., Liu F., Yang R. (2019). Targeted therapy of the AKT kinase inhibits esophageal squamous cell carcinoma growth in vitro and in vivo. Int. J. Cancer.

[30] Carvalho D.O., Freitas J., Nogueira P., Henriques S.N., Carmo A.M., Castro M.A., Guido L.F. (2018). Xanthohumol inhibits cell proliferation and induces apoptosis in human thyroid cells. Food Chem. Toxicol..

[31] Berg K.C.G., Eide P.W., Eilertsen I.A., Johannessen B., Bruun J., Danielsen S.A., Bjornslett M., Meza-Zepeda L.A., Eknaes M., Lind G.E. (2017). Multi-omics of 34 colorectal cancer cell lines—A resource for biomedical studies. Mol. Cancer.

[32] Chou T.C. (2010). Drug combination studies and their synergy quantification using the Chou-Talalay method. Cancer Res..

[33] Chou T.C. (2006). Theoretical basis, experimental design, and computerized simulation of synergism and antagonism in drug combination studies. Pharmacol. Rev..

[34] Traganos F., Ardelt B., Halko N., Bruno S., Darzynkiewicz Z. (1992). Effects of genistein on the growth and cell cycle progression of normal human lymphocytes and human leukemic MOLT-4 and HL-60 cells. Cancer Res..

[35] Hosokawa N., Hosokawa Y., Sakai T., Yoshida M., Marui N., Nishino H., Kawai K., Aoike A. (1990). Inhibitory effect of quercetin on the synthesis of a possibly cell-cycle-related 17-kDa protein, in human colon cancer cells. Int. J. Cancer.

[36] Zi X., Grasso A.W., Kung H.J., Agarwal R. (1998). A flavonoid antioxidant, silymarin, inhibits activation of erbB1 signaling and induces cyclin-dependent kinase inhibitors, G1 arrest, and anticarcinogenic effects in human prostate carcinoma DU145 cells. Cancer Res..

[37] Pagano M., Pepperkok R., Lukas J., Baldin V., Ansorge W., Bartek J., Draetta G. (1993). Regulation of the cell cycle by the cdk2 protein kinase in cultured human fibroblasts. J. Cell Biol..

[38] Delmas D., Passilly-Degrace P., Jannin B., Malki M.C., Latruffe N. (2002). Resveratrol, a chemopreventive agent, disrupts the cell cycle control of human SW480 colorectal tumor cells. Int. J. Mol. Med..

[39] Kuwajerwala N., Cifuentes E., Gautam S., Menon M., Barrack E.R., Reddy G.P. (2002). Resveratrol induces prostate cancer cell entry into s phase and inhibits DNA synthesis. Cancer Res..

[40] Heiss E.H., Schilder Y.D., Dirsch V.M. (2007). Chronic treatment with resveratrol induces redox stress- and ataxia telangiectasia-mutated (ATM)-dependent senescence in p53-positive cancer cells. J. Biol. Chem..

[41] Young L.F., Martin K.R. (2006). Time-dependent resveratrol-mediated mRNA and protein expression associated with cell cycle in WR-21 cells containing mutated human c-Ha-Ras. Mol. Nutr. Food Res..

[42] Shaltiel I.A., Krenning L., Bruinsma W., Medema R.H. (2015). The same, only different—DNA damage checkpoints and their reversal throughout the cell cycle. J. Cell Sci..

[43] Hanahan D., Weinberg R.A. (2011). Hallmarks of cancer: The next generation. Cell.

[44] Zhang J., Dai Q., Park D., Deng X. (2016). Targeting DNA Replication Stress for Cancer Therapy. Genes (Basel).

[45] Bouwman P., Jonkers J. (2012). The effects of deregulated DNA damage signalling on cancer chemotherapy response and resistance. Nat. Rev. Cancer.

[46] Wang H., Zhang X., Teng L., Legerski R.J. (2015). DNA damage checkpoint recovery and cancer development. Exp. Cell Res..

[47] Liu X., An L.J., Li Y., Wang Y., Zhao L., Lv X., Guo J., Song A.L. (2019). Xanthohumol chalcone acts as a powerful inhibitor of carcinogenesis in drug-resistant human colon carcinoma and these effects are mediated via G2/M phase cell cycle arrest, activation of apoptotic pathways, caspase activation and targeting Ras /MEK/ERK pathway. J. BUON Off. J. Balk. Union Oncol..

[48] Logan I.E., Miranda C.L., Lowry M.B., Maier C.S., Stevens J.F., Gombart A.F. (2019). Antiproliferative and Cytotoxic Activity of Xanthohumol and Its Non-Estrogenic Derivatives in Colon and Hepatocellular Carcinoma Cell Lines. Int. J. Mol. Sci..

[49] Ramesh M., Ahlawat P., Srinivas N.R. (2010). Irinotecan and its active metabolite, SN-38: Review of bioanalytical methods and recent update from clinical pharmacology perspectives. Biomed. Chromatogr..

[50] Ambroz M., Lnenickova K., Matouskova P., Skalova L., Bousova I. (2019). Antiproliferative Effects of Hop-derived Prenylflavonoids and Their Influence on the Efficacy of Oxaliplatine, 5-fluorouracil and Irinotecan in Human ColorectalC Cells. Nutrients.

[51] Miranda C.L., Stevens J.F., Ivanov V., McCall M., Frei B., Deinzer M.L., Buhler D.R. (2000). Antioxidant and prooxidant actions of prenylated and nonprenylated chalcones and flavanones in vitro. J. Agric. Food Chem..

[52] Gerhauser C. (2005). Broad spectrum anti-infective potential of xanthohumol from hop (*Humulus lupulus* L.) in comparison with activities of other hop constituents and xanthohumol metabolites. Mol. Nutr. Food Res..

[53] Legette L.L., Luna A.Y., Reed R.L., Miranda C.L., Bobe G., Proteau R.R., Stevens J.F. (2013). Xanthohumol lowers body weight and fasting plasma glucose in obese male Zucker fa/fa rats. Phytochemistry.

[54] Dorn C., Massinger S., Wuzik A., Heilmann J., Hellerbrand C. (2013). Xanthohumol suppresses inflammatory response to warm ischemia-reperfusion induced liver injury. Exp. Mol. Pathol..

[55] Jiang C.H., Sun T.L., Xiang D.X., Wei S.S., Li W.Q. (2018). Anticancer Activity and Mechanism of Xanthohumol: A Prenylated Flavonoid from Hops (*Humulus lupulus* L.). Front. Pharmacol..

[56] Pichler C., Ferk F., Al-Serori H., Huber W., Jager W., Waldherr M., Misik M., Kundi M., Nersesyan A., Herbacek I. (2017). Xanthohumol Prevents DNA Damage by Dietary Carcinogens: Results of a Human Intervention Trial. Cancer Prev. Res..

[57] Dietz B.M., Kang Y.H., Liu G., Eggler A.L., Yao P., Chadwick L.R., Pauli G.F., Farnsworth N.R., Mesecar A.D., van Breemen R.B. (2005). Xanthohumol isolated from Humulus lupulus Inhibits menadione-induced DNA damage through induction of quinone reductase. Chem. Res. Toxicol..

[58] Ferk F., Huber W.W., Filipic M., Bichler J., Haslinger E., Misik M., Nersesyan A., Grasl-Kraupp B., Zegura B., Knasmuller S. (2010). Xanthohumol, a prenylated flavonoid contained in beer, prevents the induction of preneoplastic lesions and DNA damage in liver and colon induced by the heterocyclic aromatic amine amino-3-methyl-imidazo[4,5-f]quinoline (IQ). Mutat. Res..

